# Phenotypes induced by NM causing α-skeletal muscle actin mutants in fibroblasts, Sol 8 myoblasts and myotubes

**DOI:** 10.1186/1756-0500-2-40

**Published:** 2009-03-10

**Authors:** Drieke Vandamme, Ellen Lambert, Davy Waterschoot, Davina Tondeleir, Joël Vandekerckhove, Laura M Machesky, Bruno Constantin, Heidi Rommelaere, Christophe Ampe

**Affiliations:** 1 Department of Biochemistry, Faculty of Medicine and Health Sciences, Ghent University, A. Baertsoenkaai 3, Gent, Belgium; 2 Department of Medical Protein Research, VIB, A. Baertsoenkaai 3, Gent, Belgium; 3 Department of Plant Production, Faculty of Bioscience Engineering, UGent, Coupure associations 653, Gent, Belgium; 4 CRUK Beatson Institute for Cancer Research, Garscube Estate, Switchback Rd., Bearsden, Glasgow, G63 9AE, UK; 5 Institut de Physiologie et Biologie Cellulaire, UMR CNRS/Université de Poitiers 6187, Pôle Biologie Santé, 86022 Poitiers cedex, France; 6 Current address : Ablynx N.V., Technologiepark 4, 9052 Zwijnaarde, Belgium

## Abstract

**Background:**

Nemaline myopathy is a neuromuscular disorder characterized by the presence of nemaline bodies in patient muscles. 20% of the cases are associated with α-skeletal muscle actin mutations. We previously showed that actin mutations can cause four different biochemical phenotypes and that expression of NM associated actin mutants in fibroblasts, myoblasts and myotubes induces a range of cellular defects.

**Findings:**

We conducted the same biochemical experiments for twelve new actin mutants associated with nemaline myopathy. We observed folding and polymerization defects. Immunostainings of these and eight other mutants in transfected cells revealed typical cellular defects such as nemaline rods or aggregates, decreased incorporation in F-actin structures, membrane blebbing, the formation of thickened actin fibres and cell membrane blebbing in myotubes.

**Conclusion:**

Our results confirm that NM associated α-actin mutations induce a range of defects at the biochemical level as well as in cultured fibroblasts and muscle cells.

## Background

Nemaline myopathy (NM) is a neuromuscular disorder, characterized by muscle weakness and hypotonia. It is a disease of the skeletal muscle sarcomere and is caused by mutations in components of the thin filament [1]; in about 20% of the cases this is in α-skeletal muscle actin (*ACTA1*, further referred to as α-actin) [2-4]. At the cellular level typical patient phenotypes are the presence of actin containing intranuclear or sarcoplasmic rod-shaped structures (nemaline rods) that may be present either in the sarcoplasm or in the nuclei [4]. Another congenital myopathy associated with ACTA1 mutations is actin myopathy, this disease is phenotypically similar to NM, and often studied together with NM, however the cellular inclusions here are actin aggregates instead of rods [4]. Curiously, the proportion of muscle fibers containing rods or aggregates does not correlate with the degree of muscle weakness [5].

To date, more than 100 α-actin mutations leading to NM or actin myopathy have been identified [6]. Given that α-actin is an essential protein for muscle function it is not surprising that mutations in this protein cause diseases (reviewed in Tondeleire et al., in press, [7]). Actin molecules require folding by the chaperones prefoldin and the cytoplasmic chaperonin CCT, to become functionally active [8-10] and have an absolute requirement for ATP to remain stable [11]. Their self assembly results in the formation of actin filaments that in muscle are part of the thin filaments and that interact with the various actin binding proteins such as α-actinin, tropomyosin and myosin in muscle. As a result, a disease-causing mutation in actin can affect one or more of these properties or functions and this heterogeneity at the biochemical level can cause mixed phenotypes and render diseases also heterogeneous at the cellular level. This was reflected in our previous reports on several congenital myopathy causing mutants [12-14] and Rommelaere et al, submitted. We discovered four different biochemical phenotypes [12] and were able to induce typical disease characteristics as rods and aggregates in fibroblasts, myoblasts and myotubes [12,14]. Additionally, we found that NM associated actin mutants induce cell membrane blebbing in differentiating myoblasts (Rommelaere et al., submitted). Here we biochemically analyze 12 new α-actin mutants associated with NM and investigate their cellular phenotypes as well as further characterization of 8 mutants biochemically characterized in Costa et al. [12].

## Methods

### Construction and biochemical analysis of the α-actin CCD and CFTD causing mutants

Construction and biochemical analysis of the NM causing α-actin mutants were performed as described previously [12,14,15]. Briefly, mutants were expressed as ^35^S-labeled proteins in *in vitro *transcription translation reactions in reticulocyte lysates and analysed on native gels with or without ATP, followed by autoradiography. For band-shift assays thymosin β4, DNAseI and vitamin D binding protein were added to the reaction. Co-polymerisation assays were performed by adding WT rabbit skeletal muscle α-actin. After polymerisation, the soluble and insoluble fraction were separated by centrifugation, and the amount of mutant actin in each fraction was analysed by autoradiography.

### Cell culture and transfection

Cell culture and transfection were performed as previously described [12,14]. Briefly, NIH3T3 fibroblasts were transfected with lipofectamin 2000 (Invitrogen), according to manufacturer's protocol. Coverslips were mounted for immunofluorescence after 24 hours of transfection. Sol 8 myogenic cells [16] were seeded in 12 well plates containing thermanox coverslips (Nunc). After 24 hours the cells were transfected using jetPEI (Qbiogene) or nucleofected, using cell line nucleofector kit V (Amaxa biosystems) with the pcDNA3.1 vectors encoding N-terminally myc-tagged α-skeletal muscle actin (wild type or mutant). Coverslips were mounted for immunofluorescence after 48 hours of transfection. To promote fusion (F) of myoblasts into myotubes, the growth medium was replaced by a differentiation medium with 2% horse serum. Myotubes were immunostained on day F+4 or F+5.

### Immunological staining

Standard immunological staining was performed [12,14]. To visualise the mutant actins we used a polyclonal anti-myc antibody (Abcam) and fluorescent phalloidin (Molecular Probes) for filamentous actin. The immunolabelled samples were examined using an Olympus IX71 epifluorescence microscope and images were acquired using a cooled Spot Camera (Diagnostic Instruments) and Analysis software (Soft Imaging Systems).

For statistics of stress fibre incorporation and diffuse cytoplasmic myc-actin staining, pictures were visually inspected and blindly scored by two persons. P-values were calculated by a Fisher's exact test  on the separate experiments.

## Results

### Biochemical characterization of α-skeletal muscle actin mutants causing NM

In a previous study [12], we biochemically characterized a set of actin mutants causing nemaline myopathy and discovered four different biochemical phenotypes: folding defective mutants, unstable mutants, mutants with reduced copolymerisation capacity and mutants without defects in these tests (for details and limitations of these assays see [12,15,17]). In the present study, we investigated twelve new NM mutants using similar approaches. In patient muscles, these mutants induce different abnormal structures such as intranuclear and cytoplasmic rods and/or an excess of thin filaments (Table 1).

**Table 1:** Description and results of the biochemical characterization of 16 new nemaline myopathy causing α-skeletal muscle actin mutants.

**Mutant**	**phenotype**	**Folding**	**CAP**	**ABP**	**copol**	**In actin structure (Kabsch)**	**reference**
**WT**		+	+	+	++		
**D25N**	severe NEM ┼	+	+	+	++	Subdomain I, Close to ATP cleft	[4]
**V35L**	severe NEM	+	+	+	++	Subdomain II, Buried	[19]
**P38L**	severe NEM	+	+	+	+	Subdomain II	[19]
**H73L**	severe NEM ┼	+	-	+, VDBP+/-	+	Subdomain II, In ATP cleft, stabilizing interaction with D179	[19]
**I75L**	severe NEM	+	+	+, VDBP+/-	++	Subdomain II, In ATP cleft	[19]
**E83K**	Typical NEM	+/-	+	+	+ more aggr	Helix	[19]
**R116H**	Severe NEM ┼	+ +/-	+	+	+ more aggr	Subdomain I, Helix, close to ATP cleft	[4]
**V163M**	IRM	+/-	+	+	+ more aggr	Subdomain III, Buried, near ATP-cleft	[27]
**Q246R**	mild, typical and severe	+	+	+	++	Subdomain IV, Close to F-actin contact	[19,20]
**G251D**	severe NEM	+	+/-	+	++	Subdomain IV, At surface	[19]
**R256H**	severe NEM	+	-	+	+	Subdomain IV, Close to F-actin contact	[2]
**M269R**	mild NEM	+	+	+	+	Subdomain IV, F-actin contact, hydrophobic plug	[21]

The properties investigated here are folding, binding to cyclase associated protein (CAP), binding to the actin binding proteins (ABP) thymosinβ4, DNAseI and Vitamin D binding protein (VDBP) and copolymerisation with rabbit α-skeletal muscle actin (copol). Also the location in the actin structure according to Kabsch et al. [18] is indicated, in addition to the patients phenotype as presented in the reference with NEM = nemaline myopathy, sarcoplasmic nemaline bodies; IRM = intranuclear rod myopathy.

**Figure 1:**
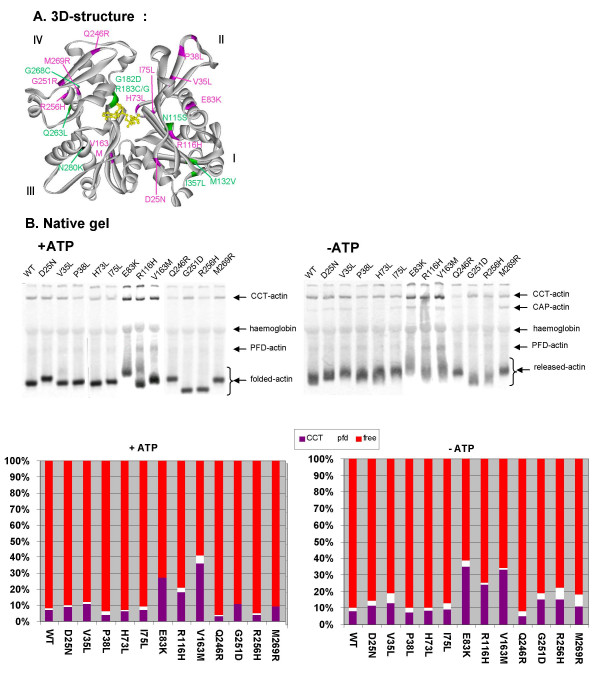
**(A) Location of the studied ACTA1 mutations on the 3D-representation of the actin molecule **[18]. Mutations indicated in green were biochemically analysed in this study, mutations indicated in pink were analysed in Costa et al. [12]. (B) Native gel analysis of twelve α-actin mutants causing NM. Autoradiograms of native gels of ^35^S-labeled WT α-actin and 12 mutants causing NM run with or without ATP. Actins were produced in *in vitro *transcription translation reactions in reticulocyte lysate, which endogenously contains CAP [22] and the actin folding machinery, prefoldin (PFD) and CCT [8,9,15]. The positions of these complexes with actin are indicated. The graph underneath indicates the percentage of actin in each of the bands (associated with CCT (purple), with prefoldin (white, (+ATP)) or CAP (white, (-ATP)) and free actin (red). In each lane the total actin is the sum of these three actin species.

The locations of the mutations in the actin molecule are displayed in pink on the 3D-representation of the actin molecule (Figure 1A) [18]. Mutations are found in subdomains I (D25N and R116H), II (V35L, P38L, H73L and I75L), III (V163M) and IV (Q246R, G251R, R256H and M269R). Some of these residues (D25, H73, I75, R116 and V163) are in or near the nucleotide-binding pocket. [19-21]

All these actin mutants are capable of folding properly, since they migrate at a position similar to monomeric actin in gels with ATP (Figure 1B) and since they can bind to the ABPs tested (i.e. thymosin β4, DNAseI and VDBP, Table 1). However, for three of the mutants act E83K, R116H and V163M a significant proportion of the proteins remains bound to CCT on native gels (Figure 1B), indicating that they fold less well compared to WT-α-actin. On gels without ATP, mutants act E83K, V163M and M269R show a slight increase in CAP-binding [22]. This effect is, however, not very strong and precludes to conclusively decide that these mutants are unstable due to impaired nucleotide binding [10,17]. We note however that mutants E83K and V163M induced more aggregation in the in vitro translation reaction than does mutant R116H (Table 1). In copolymerisation tests mutants act P38L, H73L, E83K, R116H, V163M, R256H and M269R displayed reduced copolymerization capacity, whereas mutants act D25N, V35L, I75L, Q246R and G251D show normal copolymerisation.

### Cellular phenotypes induced by the NEM causing α-skeletal muscle actin mutants

Since we previously observed that expression of myc-tagged α-actin mutants associated with NM can induce a range of defects in the actin cytoskeleton, we constructed all of these mutants with an N-terminal myc-tag and transfected them in NIH3T3 fibroblasts and Sol8 myoblasts [12,14] and Rommelaere et al. submitted. In order to analyse their incorporation in the actin cytoskeleton or the occurrence of rods or aggregates, we performed immunological staining of the myc-tagged mutants, together with fluorescent phalloidin staining for F-actin containing structures. We performed a similar analysis on myc-tagged actin mutants N115S, G182D, R183G, R183C, Q263L and G268C (green in Figure 1A), which were previously biochemically characterized in Costa et al. (Table 2) (additionally, G182D which was only characterized in fibroblasts in Costa et al. was analysed here in muscle cells) [12]. The results of this *ex vivo *analysis are summarized in Table 3.

**Table 2:** clinical phenotype and position or the function in the actin structure of mutations which were biochemically characterized in Costa *et al*. 2004 [12].

**Mutation**	**Phenotype**	**Position or Function in Actin Structure**
**N115S**	typical, NEM	Buried, near nucleotide cleft
**M132V**	mild, NEM	Buried
**G182D**	typical, NEM	in nucleotide cleft
**R183C**	severe, NEM	H-bonds for nucleotide cleft closure
**R183G**	typical./severe, NEM	H-bonds for nucleotide cleft closure
**Q263L**	severe, NEM	Surface, near hydrophobic plug
**G268C**	mild/typical. NEM	F-actin contact, hydrophobic pocket
**I357L**	Severe, NEM, IRM	buried

NEM = nemaline myopathy, sarcoplasmic nemaline bodies; IRM = intranuclear rod myopathy.

**Table 3:** Summary of the phenotypes observed upon expression of NM associated α-actin mutants in cell cultures.

**Mutant**	**Clinical phenotype**	**Cell line**	**aggreg**	**Rods**	**Fibers**/ **Cables**	**Diffuse cyto local**	**Blebs or membrane protrusions**
**WT**	-	fibroblast			X		
		myoblast			X		
		myotube			X		

**D25N**	Severe NEM ┼	fibroblast	X		X	+/-	
		myoblast	X		X		
		myotube	X		X		

**V35L**	Severe NEM	fibroblast			X	X	
		myoblast			X	X	
		myotube	X		X		

**P38L**	Severe NEM	fibroblast			X	X	
		myoblast	X		X		X
		myotube			X		

**H73L**	Severe NEM ┼	fibroblast		Cyto	X	X	
		myoblast			X		
		myotube			X		

**I75L**	Severe NEM	fibroblast		IR	X		
		myoblast	X		X		
		myotube			X	X	

**E83K**	Typical NEM	fibroblast	X		X		
		myoblast			Few	X	X
		myotube			X		

**N115S**	Typical NEM	fibroblast			X	+/-	
		myoblast			X		
		myotube			X		

**R116H**	Severe NEM ┼	fibroblast	X	Cyto, perinuclear	X		
		myoblast			Few	X	X
		myotube			X		X

**M132V**	mild NEM	fibroblast			X		
		myoblast	X		X		
		myotube			X		

**V163M**	IRM	fibroblast	X	IR + cyto	Low incorporation	+/-	
		myoblast	X	IR	Few	X	
		myotube			Thick fibers		

**G182D**	typical NEM	fibroblast			Low incorporation	+/-	
		myoblast			X	X	
		myotube			X		

**R183C**	Severe NEM ┼	Fibroblast		IR	X		X
		Myoblast	X		X		X
		Myotube			X		

**R183G**	Int + Severe NEM ┼	Fibroblast	X		X	X	
		Myoblast			X		
		Myotube			X		

**Q246R**	Mild, typical and severe NEM	Fibroblast	X		X		
		Myoblast			X		X
		Myotube			X		

**G251D**	Severe NEM	Fibroblast	X		X	+/-	
		Myoblast	X		X	X	
		Myotube			X		

**R256H**	Severe NEM	Fibroblast			X	+/-	
		Myoblast	X		X		
		Myotube			X		

**Q263L**	Severe NEM	fibroblast			X		
		myoblast	X		X		
		myotube			X		

**G268C**	Mild/typical NEM	fibroblast	X		X	+/-	
		myoblast			X	X	
		myotube			X		

**M269R**	Mild NEM	fibroblast	X		X	+/-	
		myoblast			X		
		myotube	X		X		

**I357L**	IRM ┼	fibroblast			Low incorporation	+/-	
		myoblast			X		
		myotube			X		

NEM nemaline myopathy, IRM: Intranuclear Rod Myopathy, IR intranuclear rods, cyto: cytoplasmic, a cross indicates that the phenotype or normal property (fibers/cables) is present, Int: intermediate ┼ lethal phenotype

#### α-actin mutants induce nemaline rod-like structures and aggregates in fibroblasts and myoblasts

**Figure 2:**
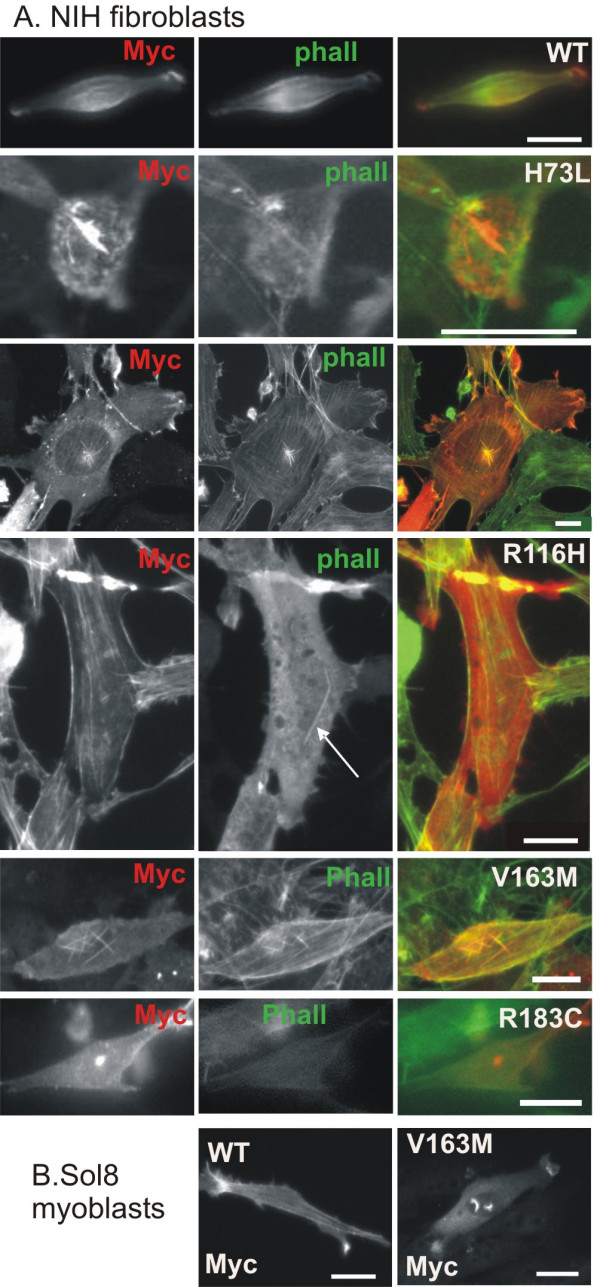
**α-actin mutants induce rods in cell line cultures**. (A) Myc-actin (red) and phalloidin (green) staining of NIH3T3 fibroblasts and (B) myc-actin staining of Sol8 myoblasts expressing WT actin or the indicated NM associated α-actin mutant. Scale bars are 20 μm.

Four of the investigated α-actin mutants induce rod-like structures in fibroblasts: I75L and V163M induce phalloidin positive intranuclear rods (Figure 2) and H73L induces cytoplasmic phalloidin positive rods (Figure 2). R116H induces perinuclear cytoplasmic phalloidin negative rods (Figure 2).

Upon expression of act V163M in myoblasts we also observed intranuclear rods. Another mutation at this position, V163L, similarly induces intranuclear rods in both fibroblasts and myoblasts [12]. V163 lies close to the nuclear export signal [23] and therefore this mutation could interfere with nuclear export resulting in intranuclear rods (Figure 2). Domazetovska *et al*. expressed EGFP- or untagged V163L and V163M actin in fibroblast and myoblast cell lines and observed similar phalloidin positive rods. Our co-polymerization results support their observation that intranuclear rods induced by V163M might be due to an increased availability of monomeric actin caused by reduced incorporation of the mutant in actin filaments [24,25]. However we can not generalize this to the other mutants as I75L for instance displays normal co-polymerisation.

**Figure 3:**
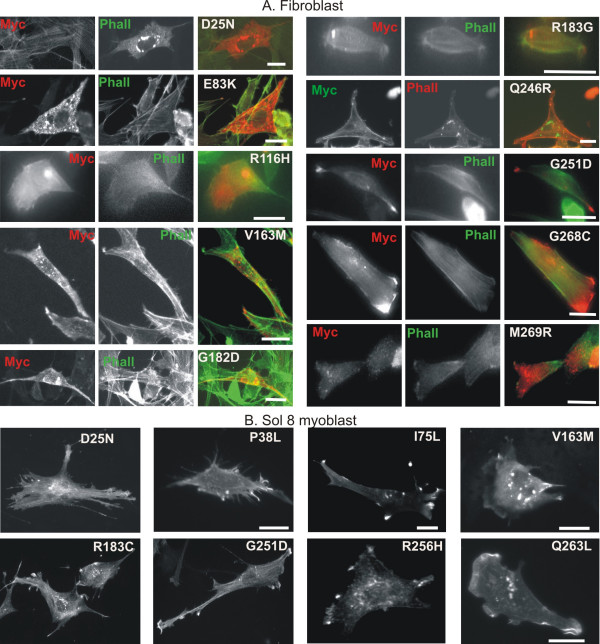
**α-actin mutants induce aggregates in cell line cultures**. (A) Myc-actin (red) and phalloidin (green) staining of NIH3T3 fibroblasts and (B) myc-actin staining of Sol8 myoblasts expressing WT actin or the indicated NM associated α-actin mutant. Scale bars are 20 μm.

Another phenotype associated with expression of nemaline myopathy associated actin mutants in cultured cells is the formation of actin-containing phalloidin negative aggregates. For eleven of the investigated mutants we observed these in fibroblasts (D25N, E83K, R116H, V163M, R183G, R183C, Q246R, G251D, G268C and M269R) (Figure 3). In most cases the aggregates were cytoplasmic or perinuclear, yet for R116H and V163M we additionally observed intranuclear aggregates, consistent with the fact that these mutants also induce intranuclear rods. Strikingly, E83K, R116H and V163M also aggregate in the *in vitro *translation assays (see above). In addition, for a subset of mutants, we also observed cytoplasmic aggregates in Sol8 myoblasts. This is the case for mutants D25N, P38L, I75L, M132V, V163M, R183C, G251D, R256H and Q263L (Figure 3).

As in previous reports [12], not all mutants induced the formation of rods or aggregates, and these phenotypes are induced more in fibroblasts than in muscle cells. This supports the hypothesis that rods can be considered secondary effects in patient muscles, which serve as 'rescue' mechanisms to remove the mutant actin from the active pool, and that other defects could underlie the muscle weakness in NM patients [26] (Rommelaere submitted).

The fact that we observed both phalloidin positive and negative rod-like structures and aggregates, is consistent with previous reports on fibroblasts and muscle cells expressing NM causing actin mutants [13,27]. A lack of phalloidin staining could indicate that the rods do not contain filamentous actin, or that the F-actin is not accessible for the phalloidin. At present it is not known whether patient rods stain with phalloidin however our results indicate that rods induced by different mutants could have a different composition and/or organisation.

#### Diffuse cytoplasmic myc-staining in fibroblasts

**Figure 4:**
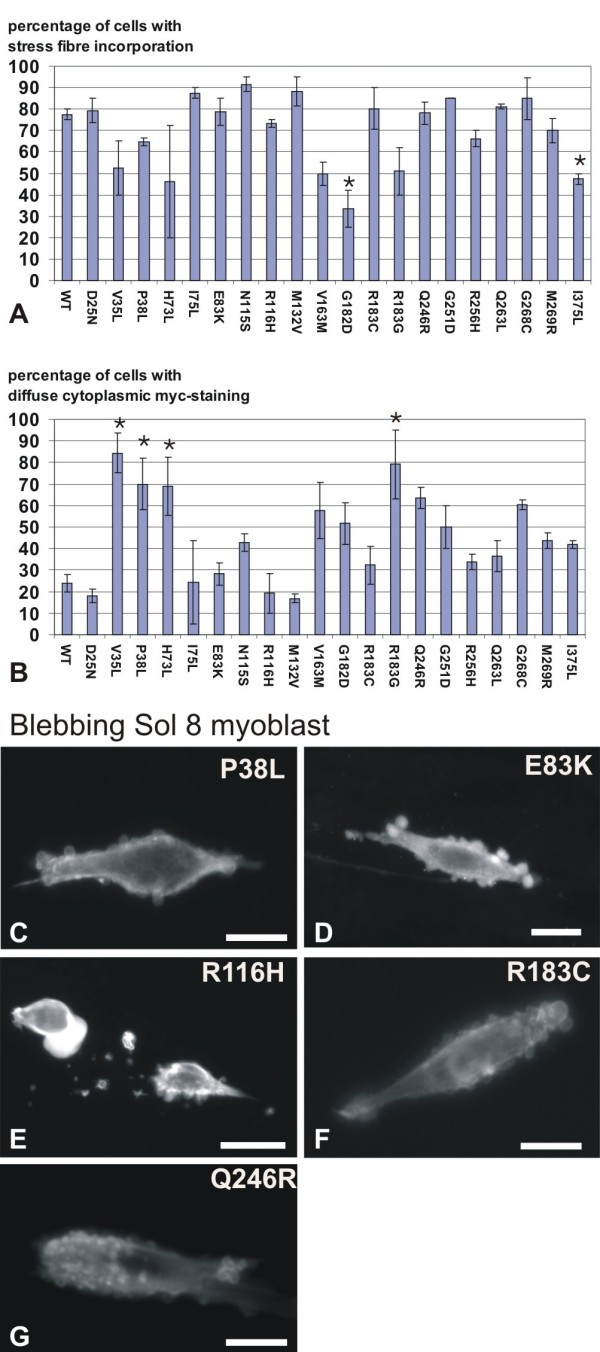
**Some α-actin mutants induce a diffuse cytoplasmic myc-staining, five α-actin mutants induce blebbing**. (A-B) graphs indicating the mean percentage of fibroblasts where myc α-actin is incorporated into stress fibres (A) or with diffuse cytoplasmic myc-actin staining (B). Error bars show the standard error of the mean. (* p < 0,05 for both measurements, or one measurement if p < 0,07 for the second measurement) (C-G) Myc-actin staining of Sol8 myoblasts expressing α-actin mutants that induce cell membrane blebbing.

To test the ability of the mutants to incorporate in filamentous structures, we calculated the percentage of fibroblasts in which a particular mutant is able to incorporate in stress fibres. In addition, we also scored for diffuse cytoplasmic staining of the myc-tagged mutant, which gives a second indication of the (lack of) incorporation in filamentous structures. Cells were visually inspected and blindly scored for both parameters by two persons and the mean was calculated. Similar to WT, all mutants are able to incorporate in stress fibres in a certain percentage of the cells. For G182D and I375L actin expressing fibroblasts this was significantly lower than for WT actin expressing cells (Figure 4A). The percentage of cells with a diffuse cytoplasmic myc-staining, shows more variation (Figure 4B): V35L, P38L, H73L, and R183G induce this cytoplasmic staining in significantly more cells than WT myc-actin. N115S, V163M, G182D, Q246R, G251D, R256H, M269R and I375L also have a tendency to display this phenotype more than cells expressing WT myc-actin, yet they do so to a lesser extent than the former mutants.

#### Five mutants induce cell blebbing in muscle cells

Consistent with our previous observation that particular NM actin mutants induce blebbing in myoblasts (Rommelaere et al., submitted), we also found five mutants here displaying this phenotype: act P38L, E83K, R116H, R183C and Q246R (Figure 4C–F). Each of these mutants is enriched in the blebs.

#### Phenotypes induced in myotubes

**Figure 5:**
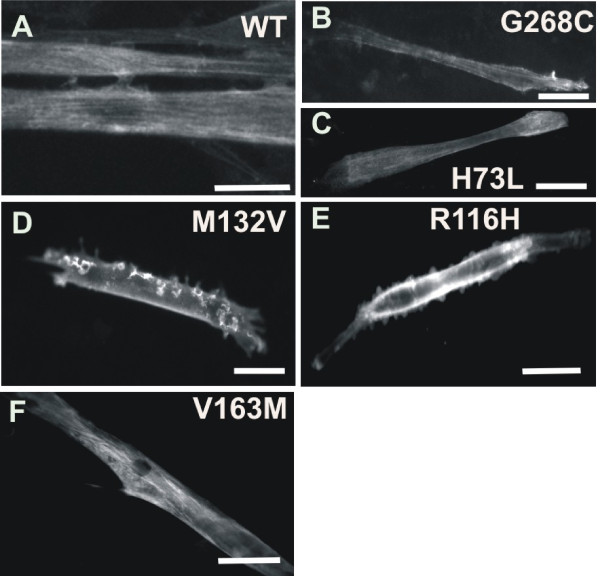
**α-actin mutants can induce phenotypes at developing myotube stage**. Myc-staining of myotube expressing (A) WT actin (B) α-actin G268C and (C) α-actin H73L show actin fibres, (D) α-actin M132V displays membrane protrusions and (E) α-actin R116H displays membrane blebbing (F) α-actin V163M has thickened actin fibres. Scale bars are 50 μm.

We differentiated myoblasts, transfected with the α-actin mutants, during five days and allowed them to form multinucleated myotubes. Immunological staining revealed that all studied mutants incorporate to a certain degree in actin fibers in myotubes (shown for H73L and G268C in Figure 5).

Act R116H, M132V, V163M transfected myotubes show an aberrant phenotype at this differentiation stage (Figure 5). In myotubes expressing act V163M, actin fibers appear thickened, a phenotype we, and others, previously also observed for act V163L (Rommelaere et al., submitted, [13]). This cellular phenotype is also consistent with the sarcomeric disorganization in *Drosophila *muscles expressing V163M [25]. Act R116H and M132V expressing myotubes display blebs or membranous protrusions as described for other NM mutants by Rommelaere et al. (Figure 5).

## Conclusion

We corroborate that NM associated α-actin mutations can induce a range of defects, both at the biochemical and cellular level. We show that the twenty NM mutants investigated here score at least one of the previously described cellular NM or actin myopathy associated phenotypes (Table 3), i.e. the cells display intranuclear or cytoplasmic rods, aggregates, blebs (only in differentiating myoblasts), diffuse cytoplasmic myc-actin staining or thickened actin fibres in myotubes. As observed before, not a single mutant can reproduce all phenotypes although some mutants display multiple, occasionally cell line dependent, phenotypes. On the other hand, not all mutants induce rod-like structures or aggregates, hallmarks in patient muscles, which supports the fact that rods could be secondary effects, and that other mutant induced defects are underlying the muscle weakness in NM patients. Phalloidin staining of cells also indicate that nemaline rods induced by different actin mutants can have different organisation or composition. Additionally, as concluded before in Costa et al., [12] there is no correlation between the observed biochemical and cellular phenotypes nor between one of these and the clinical situation.

## Competing interests

The authors declare that they have no competing interests.

## Authors' contributions

DV contributed to the immunostainings and microscopic analysis and drafting of the manuscript. HR contributed to the biochemical assays and immunostainings, together with DW. DT helped with the analysis of the stress fibre incorporation and diffuse cytoplasmic myc-staining. CA conceived the study, participated in its design and coordination and drafted the manuscript. JV, LM, BC helped drafting the manuscript and gave scientific support. All authors read and approved the final manuscript.
